# A Multidisciplinary Approach to Evaluate the Presence of Hepatic and Cardiac Abnormalities in Patients with Post-Acute COVID-19 Syndrome—A Pilot Study

**DOI:** 10.3390/jcm10112507

**Published:** 2021-06-06

**Authors:** Felix Bende, Cristina Tudoran, Ioan Sporea, Renata Fofiu, Victor Bâldea, Radu Cotrău, Alina Popescu, Roxana Sirli, Bogdan Silviu Ungureanu, Mariana Tudoran

**Affiliations:** 1 Department of Gastroenterology and Hepatology, University of Medicine and Pharmacy “Victor Babes” Timisoara, 300041 Timisoara, Romania; bende.felix@umft.ro (F.B.); isporea@umft.ro (I.S.); renata.fofiu@yahoo.com (R.F.); victorbaldea07@gmail.com (V.B.); cotrau.radu@yahoo.com (R.C.); alinamircea.popescu@gmail.com (A.P.); roxanasirli@gmail.com (R.S.); 2 Center of Advanced Research in Gastroenterology and Hepatology, Faculty of Medicine, University of Medicine and Pharmacy “Victor Babes” Timisoara, 300041 Timisoara, Romania; 3 County Emergency Hospital “Pius Brinzeu”, L. Rebreanu, Nr. 156, 300723 Timisoara, Romania; tudoran.mariana@umft.ro; 4 Center of Molecular Research in Nephrology and Vascular Disease, Faculty of Medicine, University of Medicine and Pharmacy “Victor Babes” Timisoara, E. Murgu Square, Nr. 2, 300041 Timisoara, Romania; 5 Department VII, Internal Medicine II, Discipline of Cardiology, University of Medicine and Pharmacy “Victor Babes” Timisoara, E. Murgu Square, Nr. 2, 300041 Timisoara, Romania; 6 Research Center of Gastroenterology and Hepatology, University of Medicine and Pharmacy of Craiova, 200349 Craiova, Romania; boboungureanu@gmail.com

**Keywords:** COVID-19, inflammation, cytokine storm, cardiovascular alterations, liver injury, transthoracic echocardiography, liver elastography

## Abstract

(1) Background: Patients suffering from the novel coronavirus 2019 (COVID-19) disease could experience several extra-pulmonary involvements, including cardiovascular complications and liver injury. This study aims to evaluate the presence of cardiac and liver alterations in patients with post-acute COVID-19 syndrome using transthoracic echocardiography (TTE) and liver elastography (LE). (2) Methods: A total of 97 subjects recovering from COVID-19, attending the hospital’s specialized outpatient clinic for persisting symptoms at 3 to 11 weeks after the acute illness, were included in this study. They all had a basal COVID-19 assessment, and subsequently, a clinical evaluation, laboratory tests, TTE, and LE. (3) Results: considering the presence of pulmonary injury during COVID-19, patients were divided into two groups. Although none of them had altered systolic function, we evidenced pulmonary hypertension, diastolic dysfunction, increased liver stiffness, viscosity, and steatosis in around one-third of the patients, with significantly higher values in subjects with pulmonary injury compared to those without. (4) Conclusion: persisting symptoms characterizing the post-acute COVID-19 syndrome could be explained by residual cardiac and hepatic lesions, which were worse in more severe COVID-19 forms. These patients may be at risk of developing liver fibrosis and cardiac alterations and should be investigated in the first 12 weeks after the onset of the infection.

## 1. Introduction

Coronavirus-19 (COVID-19) has erupted as a fast-spreading infectious disease caused by the novel Severe Acute Respiratory Syndrome-Coronavirus-2 (SARS-CoV-2). In March 2021, the global COVID-19 pandemic has affected all continents, with over 146.8 million confirmed cases and more than 3 million confirmed deaths [1]. The outbreak of COVID-19 has inflicted major socio-economic problems, overwhelming health services worldwide and severely impacting the quality of life [2,3,4].

The majority of patients with COVID-19 infection experience mild forms of the disease, characterized by symptoms commonly described in influenza, but 20% of the infected individuals develop moderate and severe pulmonary infection requiring hospitalization, with around 5% of them imposing assistance in critical care units [5,6]. In addition to the well-known COVID-19 pulmonary lesions, extra-pulmonary injuries have also been described, especially cardiovascular (CV) ones, but several other organs, especially the liver, could be affected as well. Multiple pathophysiological mechanisms have been proposed: direct cellular injury by the virus or mediated via Angiotensin-converting enzyme 2 (ACE-2) receptors expression, multiple inflammatory responses triggered by a cytokines storm, microthrombogenesis, endothelial dysfunction or vasculitis, often aggravated by hypoxemia, in addition to drug-induced hepatic reactions and/or the exacerbation of latent chronic liver disease (CLD) [7,8,9,10]. The spectrum of CV complications is very large, the most significant being myocardial ischemia, acute myocarditis with heart failure, arrhythmias, or even pulmonary thromboembolism and right ventricular dysfunction (RVD) [11,12]. Referring to liver injury (LI), published data suggest a higher incidence in severe COVID-19 cases, but patients suffering from moderate forms could be affected as well, increasing the risk of subsequently developing liver fibrosis (LF) [8,13].

As the COVID-19 infection continued, and more and more people got infected, it became obvious that we must consider the long-term consequences of this disease [14]. Several studies have reported persisting symptoms after COVID-19 infection, their mechanisms being not completely understood, but some of them could be explained by persisting multisystem dysfunctions, especially pulmonary sequelae, CV abnormalities, but also by long-lasting LI. Recently, terms such as post-acute COVID describing the persistence of symptoms within 3 and up to 12 weeks after the acute phase, and long COVID referring to manifestations continuing even 12 weeks after having been proposed [15]. Starting from the presumption that these syndromes are explained by multisystem abnormalities, a multidisciplinary approach is required to timely diagnose the potentially significant complications before considering them as functional disorders.

The study aims to evaluate the presence of CV and LI abnormalities in patients with post-acute COVID-19 syndrome, in a multidisciplinary setting, using transthoracic echocardiography (TTE) and liver elastography (LE), and to research, to what extent, the severity of the initial pulmonary injury and/or the inflammatory response could be related to the occurrence of these alterations.

## 2. Materials and Methods

### 2.1. Study Population

**Figure 1:**
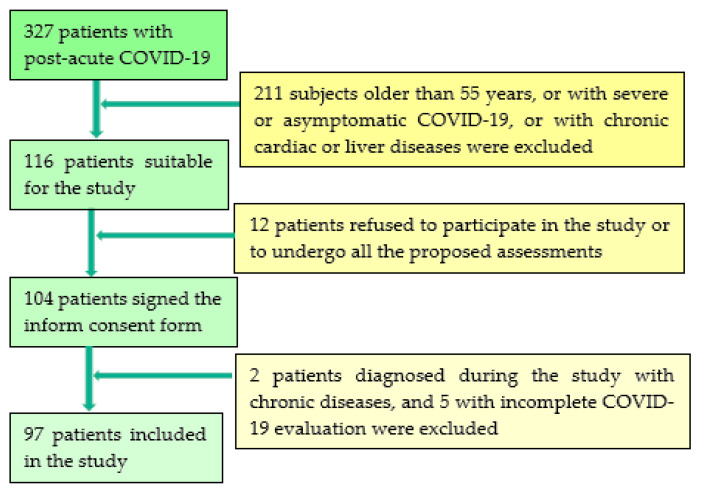
Timeline of study population selection.

In this prospective study, we included 97 consecutive subjects with post-acute COVID-19 syndrome. They were selected from 327 individuals who suffered from mild/moderate SARS-CoV-2 infection within 3 to 12 weeks and attended the specialized outpatient clinic of a major clinical and emergency hospital in western Romania between January and May 2021 for persisting symptoms such as fatigue, shortness of breath, chest discomfort, palpitations, and reduced exercise capacity, all claiming that they have not yet achieved their basal health status. In the first stage, we selected 116 patients, aged between 18 and 55, who fulfilled the inclusion/exclusion criteria. However, 12 patients refused to sign the informed consent or to undergo a cardiological or hepatological assessment. Of the remaining 104 subjects, during the study, 7 were diagnosed with significant preexisting cardiac or hepatic pathology and were excluded from the study, see Figure 1. Despite the statement from health authorities that a COVID-19 patient is considered non-infectious after 14 days following the onset of symptoms, all assessments were performed after each patient had a negative PCR test.

(a) Inclusion criteria: age ≥ 18 years old, the ability to provide informed consent; the presence of a COVID-19 infection within 3 to 12 weeks before this study, certified by a positive result of real-time reverse transcriptase–polymerase chain reaction (RT-PCR) assay of nasal and pharyngeal swabs; subjects who had a basal clinical, biological, and thoracic computed-tomographic (TCT) assessment during the acute phase of the disease; and the willingness to undergo a detailed clinical exam, TTE, and LE.

(b) Exclusion criteria: subjects confirmed with COVID-19 without a basal clinical, TCT, and laboratory assessment; those treated with antiviral therapy known to induce LI; patients with CLD of clear etiology (hepatitis C virus, hepatitis B virus, autoimmune hepatitis, primary biliary cholangitis, or primary sclerosing cholangitis); individuals with heavy alcohol consumption (ethanol intake > 210 g per week for men and >140 g per week for women), patients with ascites, patients with elevated aminotransferase levels more than five times the upper normal limit, patients with focal liver lesions, patients with severe CV disease and heart failure inducing liver congestion; subjects aged over 55 years in which age-related changes may have occurred.

After inclusion, the following data were collected from all patients: age, gender, the results of the basal COVID-19 assessment, weeks since COVID-19 confirmation, and the number of persisting COVID-19-related symptoms such as fatigue, shortness of breath, chest discomfort, palpitations, and reduced exercise capacity. All subjects underwent a complete clinical evaluation, with body mass index (BMI) assessment, laboratory tests, TTE, and liver elastography study.

The study was approved by the Research Ethics Committee and the Institutional Review Board of our University Hospital, Number 32/16 May 2019, and was performed following the World Medical Association Declaration of Helsinki, revised in 2000, Edinburgh. All the patients provided written informed consent before study entry.

### 2.2. Methods

Basal COVID-19 assessment was performed in the first days of the acute infection with the SARS-CoV2 virus, included clinical exam, laboratory findings, such as complete blood count, C-reactive protein (CRP), aspartate aminotransferase (AST), and alanine aminotransferase (ALT), as well as a TCT to evaluate the presence and severity of the pulmonary injury. TCT was performed for all subjects, and images from the acute COVID-19 were reviewed by two radiology experts for the presence of typical radiological images, such as ground-glass opacities, crazy-paving pattern, consolidation, and fibrosis, the extent of pulmonary injury being assessed based on a severity scoring. According to TCT assessment, all patients with pulmonary lesions were classified into mild (˂30% pulmonary injury) or moderate (30–60% lesions) forms [16].

All TTE examinations were performed according to guidelines recommendations [17]. After a comprehensive assessment of the cardiac morphology and function, left and right ventricular performance were evaluated, as well as diastolic dysfunction (DD), by determining the parameters described below.

Left ventricular (LV) systolic function was evaluated in 2D mode, from apical 2-, 3-, and 4-chamber view by assessing the LV ejection fraction (LVEF), by the modified Simpson formula (˂50% = pathological), and the lateral mitral annular plane systolic excursion (MAPSE), values under 10 mm being considered abnormal;

DD included the assessment of left ventricular mass index (LVMI), values ˃ 115 g/m^2^ for males and ˃95 g/m^2^ for females, defining left ventricular hypertrophy (LVH), and the measurement of left atrial volume index (LAVI), values ˃ 34 mL being considered pathologically. We registered in pulsed Doppler, at the level of the mitral valve annulus, in apical 4-chamber view, the mitral inflow, and analyzed the peak early diastolic velocity (E), the late diastolic velocity (A), and the E/A ratio. Tissue Doppler imaging, TDI, was used to record early diastolic velocity (e’) and late diastolic velocity at the level of the septal and lateral mitral annulus. Subsequently, an average and E/e’ ratio was calculated. Type I of DD was defined by an E/A ratio under 0.8 and E ˂ 50 cm/s, while type III DD was considered if the E/A ratio was over 2. In case of an E/A ratio under 0.8, but with E over 50 cm/s, or if E/A ratio was between 0.8 and 2, type II DD was suspected, which was certified if at least two of the following three criteria were present: average E/e’ ˃ 14, LAVI ˃ 34 mL/m^2^, and/or TRV ˃ 2.8 m/s. In cases where only one of the three previously mentioned criteria was fulfilled, a type I DD was diagnosed [18].

Right ventricular (RV) dysfunction RVD was evaluated in 4-chamber view, by determining the fractional area change (FAC-values ˂ 35% being pathological) and the tricuspid annular plane systolic excursion (TAPSE), assessed in M-mode at the level of the lateral tricuspid valve annulus (values ˂ 17 mm defining RVD).

Systolic pressure in the pulmonary artery (sPAP) was estimated in continuous-wave Doppler, from the apical window at the level of the tricuspid valve, based on the assessment of peak tricuspid regurgitation velocity (TRV), by taking into account the right atrial pressure, determined by measuring the inferior vena cava diameter and its respiratory variations. In this study, we considered that sPAP values of ≥35 mmHg at rest indicate PH with severity ranging from mild (35–44 mmHg) to moderate (45–60 mmHg) to severe (>60 mmHg) [19,20].

Patients were examined using two different elastography techniques; initially, they were evaluated using the Aixplorer MACH^®^ 30 ultrasound system (SuperSonic Imagine, Aix-en-Provence, France), and afterward, Transient Elastography (TE) and Controlled Attenuation Parameter (CAP) measurements were performed with FibroScan^®^ Compact 530 (EchoSens, Paris, France).

Liver stiffness, steatosis, and viscosity were assessed using Shear Wave elastography (2D-SWE PLUS), Sound Speed Planewave ultrasound (SSp PLUS), Attenuation Plane-wave ultrasound (Att PLUS), and Viscosity Plane-wave ultrasound (Vi PLUS) using UltraFast™ Imaging ultrasound platform, embedded on the new Aixplorer MACH^®^ 30 system (Supersonic Imagine, Aix-en-Provence, France). In the same session, liver stiffness and steatosis were assessed using TE with CAP (FibroScan, EchoSens, Paris, France) measurements. The measurements were performed following the recommendations of the latest elastography guidelines [21], in the same session by two experienced operators, blinded to each other’s results and the patient’s clinical data.

− ShearWave PLUS Elastography

The Aixplorer Mach 30 ultrasound system, with a C6-1X convex probe, was used to perform the 2D-SWE measurements. The shear wave elasticity measurement box was placed at least 1–2 cm below the liver capsule in an area free of large vessels or artefactual areas (reverberation, noisy areas from rib shadowing). Once the SWE map was suitable, the patient was asked to hold their breath while an image acquisition was performed, and then the Q-Box™ was placed over an area of relative homogeneous elasticity, at a depth of 3–5 cm. The new Aixplorer system with UltraFast™ technology offers a new quality parameter, the measurement stability index tool (SI); therefore, each SWE measurement was acquired at a stability index > 90%. Reliable measurements were defined as the median value of five SWE measurements (obtained from five different frames) with an interquartile range interval (IQR) to the median ratio (IQR/M) < 30%.

− Viscosity PLUS

Vi PLUS is an additional parameter obtained at the same time as the SWE examination, Vi PLUS provides information on the dispersion of the tissue shear wave (analysis of the propagation speed of the shear wave at several frequencies). The changes in the speed of the shear waves between frequencies are represented both qualitatively in the form of a color-coded map as well as quantitative, expressed in pascal-second (Pa·s) over a range of values.

− Attenuation PLUS and Sound Speed PLUS

The Sound Speed Plane Wave Ultrasound (SSp PLUS) is a new technology that allows quantification of the intrahepatic speed of sound, reflecting fat content, being an indicator of hepatic steatosis. Local measurement of the sound speed is expressed in m/s over a range of values (from 1450 m/s to 1600 m/s).

The Attenuation Plane Wave Ultrasound (Att PLUS) mode provides information about tissue ultrasound beam attenuation through an ROI by measuring the decrease in amplitude of the ultrasound waves as they propagate as a function of frequency. The ultrasound beam attenuation information is quantitative. Local measurement of the tissue attenuation is expressed in dB/cm/MHz over a range of values (from 0.2 dB/cm/MHz to 1.6 dB/cm/MHz). The measurements were performed using the same transducer used for 2D-SWE evaluation following the acquisition protocol proposed by the manufacturer: with the patient in a supine position, by intercostal approach, with the probe perpendicular to the liver surface, the ROI was placed in a homogeneous area of the liver parenchyma free of large vessels, and acquisitions were initiated during neutral respiratory apnea. As recommended by the manufacturer’s whitepaper, reliable measurements were defined as the median value of five measurements, with an IQR/M < 30%.

− Transient Elastography and Controlled Attenuation Parameter

TE with CAP measurements was performed using FibroScan^®^ Compact 530, the standard M (3.5 Hz frequency) probe, or the XL (2.5 Hz frequency) probe were used as suggested by the Automatic Probe Selection tool. Reliable results, representing the median value of 10 valid measurements, with an IQR/M ≤ 30%, are expressed in kilopascals (kPa) with values ranging (min-max) between 2.5 and 75 kPa for fibrosis and in dB/m with values ranging (min-max) between 100 and 400 dB/m for steatosis [22]. A CAP cut-off value of 294 dB/m can be used to differentiate between subjects with no liver steatosis (S0) and liver steatosis (S1–S3) [23].

The statistical analysis was performed using MedCalc Version 19.4 (MedCalc Software Corp., Brunswick, ME, USA) and Microsoft Office Excel 2019 (Microsoft for Windows). Descriptive statistics were used for demographic, anthropometric, and clinical data of the patients. The distribution of numerical variables was tested using the Kolmogorov–Smirnov test and numerical variables with normal distribution are presented as means ± standard deviation, while variables with non-normal distribution are presented as median values and range. Qualitative variables are presented as percentages and numbers. Comparisons between groups were performed using the Chi-squared test for categorical variables or the Student’s *t*-test for continuous variables. A *p*-value < 0.05 was considered significant for each statistical test. The relationship between the different laboratory findings, echocardiographic, and liver elastography parameters was determined using Spearman’s correlation coefficient. Univariate and multivariate regression analysis was used to identify independent predictors for the presence of cardiac abnormalities and liver stiffness changes in COVID-19 patients.

## 3. Results

The study group included a total of 97 patients with COVID-19 infection, 37 men and 60 women, aged between 21 and 55 years, mean age 43.88 ± 9.31 years, who suffered from COVID-19 and underwent the first assessment during the acute phase of the disease, and subsequently, a second evaluation during the first 11 weeks after being confirmed with COVID-19. All included subjects underwent TTE and liver elastography using transient elastography (TE), while 84/97 (86.6%) subjects also underwent liver elastography evaluation using the Aixplorer technique (ShearWave PLUS, SSp PLUS, Att PLUS, Vi PLUS). Their demographic and clinical characteristics, as well as the results of laboratory tests, are described in Table 1, while TTE and liver elastography findings are summarized in Table 2.

According to the presence and severity of the pulmonary injury assessed on TCT at the initial evaluation, study subjects were divided into two subgroups (with and without pulmonary involvement).

In the subset of patients with pulmonary injury, 53 patients were included, 23 men and 30 women, aged between 29 and 55 years, mean age 46.01 ± 7.66 years, who suffered from COVID-19 infection with 3 and up to 10 weeks ago, all having pulmonary injury ranging from 2 to 40%, and 6 of them being hospitalized for moderate SARS-CoV-2 induced pneumonia. Of these patients, 15 were overweight (27.41 ± 1.39 kg/m^2^) and 28 were obese (BMI of 35.5 ± 4.70 kg/m^2^), of which 16 subjects had obesity class 1, 7 subjects class 2, and 5 subjects obesity class 3. In total, 25 patients had associated pathologies, 23 having systemic hypertension grade I, 4 diabetes mellitus, and 1 altered basal glycemia. The results of their TTE examinations are detailed in Table 2.

Based on TTE, we determined in 30 patients elevated values of TRV over 2.8 m/s, defining pulmonary hypertension (PH), with sPAP levels ranging from 37 to 49.89 mmHg, 17 cases with mild and 13 with moderate forms. In total, 18 patients had reduced FAC and 14 lower TAPSE, characterizing RVD. Only two patients had reduced LV function. We determined in 7 patients E/A ratio ˂ 0.8, the remaining having 0.8 ˂ E/A ˂ 2, and we evidenced in 13 of them an increased E/e′ ratio of over 14. Thus, DD of type 2 was diagnosed in 11 cases, and of type 1 in 11 subjects, taking into account that increased TRV was determined in 11 more cases with an E/A ratio between 0.8 and 2. A total of 19 individuals, 7 men and 12 women, had LV hypertrophy.

The second subset of patients, without pulmonary lesions, included 44 subjects, 14 men and 30 women, aged between 21 and 55, mean age 41.31 ± 10.49, diagnosed with COVID-19, within 5 and up to 11 weeks ago, none of them being hospitalized. In total, 19 patients had normal BMI, 16 were overweight (27.91 ± 1.29 kg/m^2^), and 9 were obese (34.41 ± 3.37 kg/m^2^), of which 6 had class 1 obesity, 2 class 2 obesity, and only 1 subject had class 3 obesity. As associated pathologies, 8 patients had systemic hypertension grade I, 3 had diabetes mellitus, and 1 had altered basal glycemia.

After the TTE assessment, we evidenced in four patients increased values of sPAP with TRV (over 2.8 m/s), none of them having reduced FAC and/or TAPSE. Only one of these cases had reduced LVEF and MAPSE. We determined in seven patients an E/A ratio ˂ 0.8 and in three subjects an elevated E/eꞌ ratio, two of them also having increased LAVI and one higher TRV. As a result, seven patients had type 1 DD and two had type 2 DD. LV hypertrophy was evidenced in five women and three men.

Patients with liver elastography evaluation were also divided according to the presence of pulmonary injury into two groups (Table 2). For LS by TE, LS by 2D-SWE SSI PLUS, and Vi PLUS, mean values were significantly higher in subjects with pulmonary injury compared to those without, while no significant differences were found between the mean values of CAP and Att PLUS. SSp PLUS mean values were significantly lower in subjects with pulmonary injury (Table 2).

**Figure 2:**
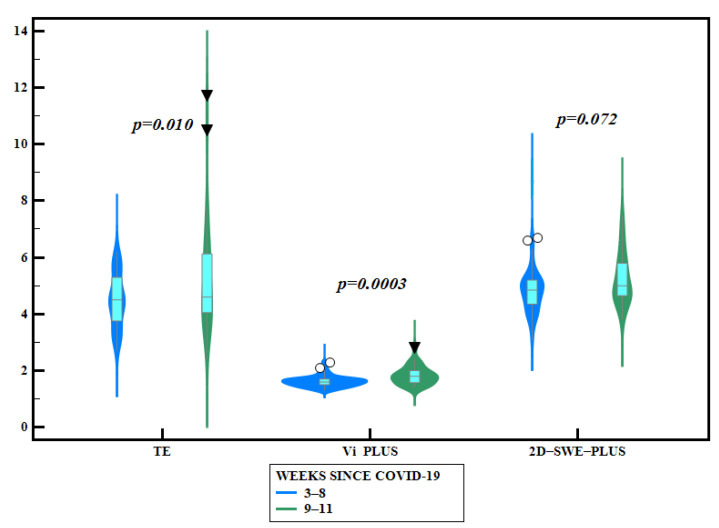
Violin plots comparing different liver elastography parameters according to the time elapsed after COVID-19 infection (blue outline plots = before 8 weeks since COVID-19 infection; green box plots = after 8 weeks since COVID-19 infection).

**Figure 3:**
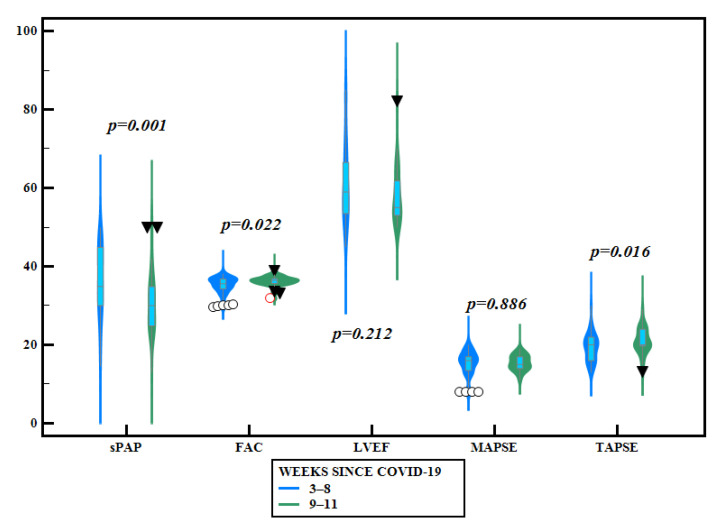
Violin plots comparing different echocardiographic parameters according to the time elapsed after COVID-19 infection (blue outline-plots = before 8 weeks since COVID-19 infection; green outline plots = after 8 weeks since COVID-19 infection).

In accordance with the time elapsed from the COVID-19 diagnosis until TTE and liver elastography evaluation, subjects were divided into two subgroups: assessments performed in the first 8 weeks—60 patients—and within 9 to 11 weeks—37 subjects. LS mean values by TE and Vi PLUS values were significantly higher in subjects evaluated in weeks 9–11 after diagnosis, compared with those evaluated earlier (5.24 ± 1.92 vs. 4.48 ± 0.95, *p* = 0.010 for TE and 1.82 ± 0.32 vs.1.61 ± 0.19, *p* = 0.0003 for Vi PLUS, respectively) (Table 3, Figure 2). TAPSE and FAC values were significantly higher in subjects evaluated in weeks 9–11 after diagnosis compared with those evaluated earlier, while TRV, sPAP, and LVEF values were significantly higher in those evaluated during the first 8 weeks (Table 3, Figure 3).

The statistical analysis by using Spearman’s correlation evidenced statistically significant correlations between the number of symptoms and the initial severity of pulmonary lesions and CRP levels (r = 0.74 and r = 0.47, respectively, *p* < 0.0001), as well as with the time since the acute phase of the COVID-19 infection (r = −0.55, *p* =< 0.0001). Similarly, statistically significant associations were detected between the severity of the initial lung injury, assessed on TCT, and CRP levels, as well as the amplitude of PAPs (r = 0.67 and r = 0.54, respectively, *p* < 0.0001), RVD assessed by FAC (r = −0.52 and r = −0.55, respectively, *p* < 0.0001), and TAPSE (r = −0.44, *p* < 0.0001 and r = −0.36, *p* = 0.0003, respectively). We identified a statistically significant correlation between the severity of pulmonary lesions and LVMI values and LS by TE (r = 0.30, *p* = 0.003), SSI SWE (r = 0.23, *p* = 0.029), CAP (r = 0.56, *p* < 0.0001), and ViPLUS (r = 0.28, *p* = 0.008).

To identify which independent factors could be responsible for the presence of cardiac abnormalities and alterations of liver elasticity in COVID-19 patients, we built a regression model in univariate regression analysis. CRP levels were independent predictors for the occurrence of cardiac abnormalities, such as higher levels of sPAP (*p* = 0.001) and lower levels of FAC (*p* = 0.013), while CRP levels were not independent predictors for liver abnormalities (*p* = 0.87 for LS by TE and *p* = 0.97 for VI PLUS values). Pulmonary injury, evaluated with TCT, was independently associated with cardiac abnormalities expressed by higher levels of sPAP (*p* < 0.001) and lower levels of FAC (*p* < 0.001), and also with liver injury defined by higher LS values by TE (*p* = 0.022). In multivariate logistic regression, the model including CRP levels (*p* < 0.0001) and the pulmonary injury, evaluated with TCT and expressed as a percentage (*p* < 0.0001), were associated only with cardiac abnormalities (higher levels of sPAP and lower levels of FAC).

## 4. Discussion

Over the past year, as the COVID-19 pandemic has spread around the globe affecting an increasing number of people, it has become obvious that there are far more health consequences than originally thought, some of them persisting a long time even after apparent recovery from the acute phase of the infection. More and more patients reported, even at 2–3 months after the acute phase of COVID-19, that they continued to suffer from a wide range of nonspecific symptoms, such as fatigue, shortness of breath, palpitations, chest pain, weakness, reduced exercise capacity, disturbed sleep, and even mental health disturbances [24]. The pathophysiological backgrounds are not completely understood, but in principle, we may consider that some patients may suffer from serious sequelae, such as pulmonary, CV, neuropsychic, or LI, while other individuals report non-specific, milder symptoms in the absence of significant organ damage [24]. The proposed pathophysiological mechanisms, such as the direct cellular effect of the virus, mediated via ACE-2 receptors expression, or indirect effects determined by inflammation or exacerbated immune responses, ischemia, and microangiothrombosis, are responsible for the multisystem damages in COVID-19, explaining both acute pulmonary injury as well as the subsequent cardiac, vascular, neuropsychological, and liver alterations [25,26,27,28]. Most commonly, CV complications, either severe, such as RVD persisting after a TEP, arrhythmias, myocardial ischemia, and HF after acute myocarditis, or mild dysfunctions, such as palpitations, sinus tachycardia, or hypotension, may explain the persistence of symptoms during recovery, but consequences of an acute LI or the exacerbation of a preexisting CLD could also be a cause.

Some authors have reported, in several medical studies, an increased prevalence of RVD observed in 39% of COVID-19 patients, while reduced EF was evidenced in 10%, and DD in 16% of them [29]. In our study, we determined an increased prevalence of PH (35.05%) similar to the literature data. This finding was of mild severity in most cases and was much more frequently diagnosed in patients with pulmonary injury during the acute phase, being frequently associated with RVD, and was seldom evidenced in subjects without pneumonia. Only 3.09% of our patients had reduced LVEF, while DD, defined by an E/e’ ratio and TRV values of over 2.8 m/s, was diagnosed in 31.95% of the subjects, many of them being overweight and/or diagnosed with systemic hypertension. However, since TRV values are used to calculate sPAP and elevated levels of over 2.8 m/s are considered a criterion for DD diagnosis, it is challenging to separate the contribution of each pathology. All these abnormalities evidenced on TTE were significantly correlated with the severity of the initial pulmonary injury and/or the level of CRP, as well as with the number of weeks since the acute phase of COVID-19 and the number of remaining symptoms.

Previous studies showed that liver damage has been identified in around 60% of patients suffering from SARS [30], and SARS-COV-2 is associated with dysfunction or damage of liver tissue [31].

In the present study, the mean liver stiffness values assessed by TE and 2D-SWE in both examined cohorts (with and without pulmonary injury) were around a value of 5 kPa, indicating a normal liver stiffness value, with no liver fibrosis [21]. Additionally, we found significantly higher mean LS values assessed by TE in COVID-19 patients with pulmonary injury compared with those without (5.08 ± 1.40 vs. 4.39 ± 1.41, *p* = 0.017), but still within the normal limits of liver fibrosis. Higher LS values were also found in COVID-19 patients with pulmonary injury using 2D SWE SSI (5.23 ± 1.00 vs. 4.79 ± 0.79, *p* = 0.028).

A significantly higher mean Vi PLUS value was found in COVID-19 patients with pulmonary injury (1.74 ± 0.28 vs. 1.64 ± 0.25, *p* < 0.0009) in our study. While inflammation plays a pivotal role in the development and progression of liver fibrosis, the non-invasive assessment of inflammation has been studied in past years, with promising results [32,33,34,35,36,37]. Several preliminary studies have shown that viscosity might be a useful parameter in tissue characterization, and it might help to detect necroinflammation in the liver [33,34]. Only a few studies have evaluated tissue viscosity as an expression of liver inflammation [32,35,36]. In the studies performed by Chen et al. and Deffieux et al., it was found that viscosity was a poor predictor not only of liver fibrosis stage, but also of disease activity and steatosis grade. These results are in contrast to the findings reported in the study performed by Sugimoto et al. [33], which found that viscosity was a good predictor of the inflammation grade and was also influenced by the steatosis grade. Based on our findings, we could speculate that patients with more severe COVID-19 disease and advanced pulmonary injury would develop an exacerbated immune response, which could lead to an increase in the dispersion slope (viscosity), whereas augmentation of fibrotic changes will mainly lead to a higher SW speed (viscoelasticity). However, more longitudinal data are required to conclude whether liver tissue viscosity is influenced by the severity of COVID-19.

The quantification of liver steatosis was performed using three different non-invasive methods (CAP, SSp PLUS, Att PLUS), and our results showed that patients with pulmonary injury had higher liver steatosis values compared to those without pulmonary injury, SSp PLUS reaching statistical significance. SSp PLUS has been recently evaluated in other studies [38,39], showing a significant negative correlation with the degree of steatosis measured by CAP (r = −70, *p* < 0.001). Additionally, LS values assessed by TE and Vi Plus values were significantly higher in patients evaluated after at least 8 weeks since COVID-19 infection diagnosis compared with those diagnosed before 8 weeks.

When comparing in our study the echocardiographic and elastographic parameters in patients evaluated before 8 weeks and after 8 weeks after the COVID-19 infection, we noticed that most of the values tended to be higher as time progresses. Although this comparison is performed between the values of different patients, it highlights the importance of a close and future follow-up of patients with post-acute COVID-19 syndrome.

The main limitation of the study is the small number of patients included. Additionally, patients’ follow-up is another important aspect, which is not presented in the current study. Another limitation is the fact that the subjects included in the study were not evaluated by liver elastography before COVID-19 infection, so the liver elastography results cannot be associated with certainty of COVID-19 infection. However, as shown in the present study, the pulmonary injury was associated as an independent factor for higher liver stiffness values. Patients with increased liver viscosity index may be at risk of developing subsequent liver fibrosis, and liver elastography follow-up is necessary. Future studies with larger cohorts of patients with liver elastography evaluation, including viscosity assessment and follow-up, are needed.

Since the number of persons suffering from post-acute COVID-19 is far from being estimated, and it is expected that more and more patients will experience long-term effects or functional symptoms, it is essential that this vulnerable patient population is cared for in a multidisciplinary approach based on comprehensive, organized systems to allow a deeper understanding of the long-term health consequences of COVID-19 on various organs and systems. Consequently, follow-up schemes and therapeutic interventions will be more efficient, resulting in improved medical assistance for a multitude of patients.

## 5. Conclusions

In patients with post-acute COVID-19 syndrome, persisting symptoms could be explained by residual cardiac and liver lesions, of which the severity is greater in more severe COVID-19 forms. These patients may be at risk of developing liver fibrosis and cardiac disease and should be investigated in this regard in the first 12 weeks after the onset of the infection.

## Figures and Tables

**Table 1:** Clinical and demographic characteristics of the included subjects according to the presence and severity of the pulmonary injury.

Demographic Characteristics, Laboratory Data	COVID-19 Patients with Pulmonary Injury (2–40%) *n* = 53	COVID-19 Patients without Pulmonary Injury *n* = 44	*p*-Value
Age (years)	46.01 ± 7.66	41.31 ± 10.49	*0.01*
Gender:			
Females	30/53 (56.6%)	30/44 (68.2%)	0.33
Males	23/53 (43.4%)	14/44 (31.8%)	0.33
BMI (kg/m^2^)	30.66 ± 6.54	26.53 ± 5.41	*0.001*
**Results of the Initial COVID-19 Assessment**			
TCT pulmonary injury	13.71% (2–40)	0%	*0.03*
Initial CRP (mg/L)	26.12 ± 15.73	18.63 ± 9.53	*0.006*
ALT (IU/L)	55.35 ± 41.24	34.95 ± 18.39	*0.007*
AST(IU/L)	44.64 ± 30.99	32.47 ± 11.36	*0.015*
O_2_ saturation (%)	96.28 ± 1.34	97.68 ± 3.62	0.07
Weeks since COVID	7 (3–10)	8 (4–11)	*0.01*
Nr. of symptoms	4 (2–6)	2 (1–5)	*˂0.0001*

Legend: BMI = body mass index; TCT = thorax computer tomography; C = Reactive Protein; ALT = alanine aminotransferase; AST = aspartate aminotransferase. Data are presented as number and percentage or mean ± standard deviation. Italic *p*-values highlight statistical significance (*p* < 0.05).

**Table 2:** TTE and liver elastography parameters according to the presence of pulmonary injury.

Parameters	COVID-19 Patients with Pulmonary Injury (2–30%), *n* = 53	COVID-19 Patients without Pulmonary Injury, *n* = 44	*p*-Value
**Echocardiographic Parameters**			
LVMI (˂95/115 g/m^2^)	97.05 ± 27.96	85.47 ± 20.45	*0.024*
LAVI (˂34 mL/m^2^)	18.51 ± 6.63	16.9 ± 7.05	0.250
E/A (˃1)	1.18 ± 0.74	1.06 ± 0.20	0.299
E/e’ (˂14)	13.0 ±2.77	12.83 ± 2.33	0.876
TRV (˂2.8 m/s)	2.89 ± 0.30	2.37 ± 0.38	*<0.0001*
sPAP (˂ 35 mmHg)	38.97 ± 6.97	28.23 ± 7.31	*<0.0001*
TAPSE (˃17 mm)	19.33 ± 3.75	20.59 ± 2.95	0.073
FAC (˃35%)	34.75 ± 1.98	36.54 ± 1.39	*<0.0001*
MAPSE (˃10 mm)	15.14 ± 2.89	15.78 ± 2.31	0.238
LVEF (˃50%)	60.0 ± 10.48	59.39 ± 7.18	0.744
**Liver Elastography Evaluation**			
LS by TE (kPa)	5.08 ± 1.40	4.39 ± 1.41	*0.017*
LS by 2D-SWE SSI PLUS (kPa)	5.23 ± 1.00	4.79 ± 0.79	*0.028*
Vi PLUS (PaS)	1.74 ± 0.28	1.64 ± 0.25	*0.0009*
CAP (db/m)	291.64 ± 71.10	266.06 ± 60.77	*0.062*
SSp PLUS (m/s)	1530.3 ± 24.91	1542.88 ± 27.66	*0.031*
Att PLUS (dB/cm/mHz)	0.47 ± 0.11	0.45 ± 0.10	0.385

Legend: LVMI = left ventricular mass index; LAVI = left atrial volume index; E/A = peak mitral inflow early (E) to late (A) diastolic velocities in pulsed Doppler; E/eꞌ = early mitral inflow diastolic velocity E to average e′ velocity (E/e′) in pulsed tissue Doppler; TRV = peak tricuspid regurgitation velocity; sPAP = systolic pressure in the pulmonary artery; TAPSE = tricuspid annular plane systolic excursion; FAC = fractional area change; MAPSE = mitral annular plane systolic excursion; LVEF = left ventricular ejection fraction; LS = liver stiffness, TE = Transient Elastography; CAP = Controlled Attenuation Parameter; 2D-SWE PLUS = Two-dimensional ShearWave PLUS Elastography by SuperSonic Imagine; SSp PLUS = Sound Speed Plane Wave Ultrasound; Att PLUS = Attenuation Plane wave ultrasound; Vi PLUS = Viscosity Plane wave ultrasound. Data are presented as mean values ± standard deviation. Italic *p*-values highlight statistical significance (*p* < 0.05).

**Table 3:** Comparison between subjects according to the time elapsed from the COVID-19 diagnosis until TTE and liver elastography.

	First 8 Weeks *n* = 60	Weeks 9–11 *n* = 37	*p*-Value
**Echocardiographic Parameters**			
E/A	1.16 ± 0.70	1.07 ± 0.19	*p = 0.447*
E/e’	12.99 ± 2.75	12.82 ± 2.28	*p* = 0.753
TRV	2.75 ± 0.42	2.50 ± 0.38	*p = 0.004*
sPAP	36.72 ± 9.08	30.63 ± 7.71	*p = 0.001*
TAPSE	19.25 ± 3.45	20.97 ± 3.21	*p = 0.016*
FAC	35.15 ± 2.16	36.22 ± 1.33	*p = 0.022*
MAPSE	15.40 ± 3.03	15.48 ± 1.92	*p* = 0.886
LVEF	60.85 ± 10.05	57.90 ± 7.04	*p = 0.212*
**Liver Elastography Evaluation**			
LS by TE (kPa)	4.48 ±0.95	5.24 ± 1.92	*p = 0.010*
LS by 2D-SWE SSI PLUS	4.87 ± 0.91 (*n* = 52)	5.24 ± 0.9 (*n* = 32)	*p* = 0.072
Vi PLUS (PaS)	1.61 ± 0.19 (*n* = 52)	1.82 ± 0.32 (*n* = 32)	*p = 0.0003*

Legend: E/A = peak mitral inflow early (E) to late (A) diastolic velocities in pulsed Doppler; E/eꞌ = early mitral inflow diastolic velocity E to average e′ velocity (E/e′) in pulsed tissue Doppler; TRV = peak tricuspid regurgitation velocity; sPAP = systolic pressure in the pulmonary artery; TAPSE = tricuspid annular plane systolic excursion; FAC = fractional area change; MAPSE = mitral annular plane systolic excursion; LVEF = left ventricular ejection fraction; LS-liver stiffness, TE = Transient Elastography; 2D-SWE PLUS = two-dimensional ShearWave PLUS Elastography by SuperSonic Imagine; Vi PLUS = Viscosity Plane-wave ultrasound. Data are presented as mean values ± standard deviation. Italic *p*-values highlight statistical significance (*p*< 0.05).

## Data Availability

Our data are available on http://dx.doi.org/10.17632/xnksp9jzgp.1.
